# Schistosomiasis Elimination: Beginning of the End or a Continued March on a Trodden Path

**DOI:** 10.3390/tropicalmed4020076

**Published:** 2019-05-05

**Authors:** Robert Bergquist, Darren J. Gray

**Affiliations:** 1 Ingerod, SE-454 94 Brastad, Sweden; 2 Research School of Population Health, Australian National University, Canberra, ACT 2601, Australia; darren.gray@anu.edu.au

**Keywords:** schistosomiasis elimination, snail control, high-sensitivity diagnostics, chemotherapy, vaccine development, health education, sanitation

## Abstract

In spite of spectacular progress towards the goal of elimination of schistosomiasis, particularly in China but also in other areas, research gaps and outstanding issues remain. Although expectations of achieving elimination of this disease have never been greater, all constraints have not been swept aside. Indeed, there are some formidable obstacles, such as insufficient amounts of drugs to treat everybody and still limited use of high-sensitive diagnostic techniques, both for the definitive and the intermediate hosts, which indicate that prevalence is considerably underrated in well-controlled areas. Elimination will be difficult to achieve without a broad approach, including a stronger focus on transmission, better diagnostics and the establishment of a reliable survey system activating a rapid response when called for. Importantly, awareness of the crucial importance of transmission has been revived resulting in renewed interest in snail control together with more emphasis on health education and sanitation. The papers collected in this special issue entitled ‘Prospects for Schistosomiasis Elimination’ reflect these issues and we are particularly pleased to note that some also discuss the crucial question when to declare a country free of schistosomiasis and present techniques that together create an approach that can show unequivocally when interruption of transmission has been achieved.

## 1. Introduction

Although severe morbidity due to schistosomiasis is disappearing, the distribution of the disease has not changed since the atlas of schistosomiasis distribution was published in 1987 [1]. Considering the population increase since then, the number of humans currently at risk must be well over a billion rather than the commonly cited figure of 800 million. According to latest available information somewhere between 230 and 250 million people are actually infected [2,3]. However, these data must be even more strongly underestimated since they are based on stool examination according to Kato-Katz [4] for intestinal schistosomiasis (basically *Schistosoma mansoni*, *S, japonicum* and *S. mekongi*) and urine filtration for the urogenital form of the disease (*S. haematobium*). Diagnosis based on the schistosome circulating cathodic antigen (CCA) finds up to four times more infected individuals compared to egg detection [5], which is supported by molecular diagnostics, e.g., the quantitative polymerase chain reaction (qPCR), a highly sensitive and specific technique [6,7]. However, the total number of infected people is not necessarily as high as four times more (as indicated by CCA) since the discrepancies reported also depend on the intensity of infection. Nonetheless, even with a multiplicator as low as two, we remain far from elimination of schistosomiasis and one wonders why control efforts for such a widespread infection have been so inadequate that the World Health Organization (WHO) lists the disease among the neglected tropical diseases (NTDs). The funds going into schistosomiasis research, however, tell another story, and we are already starting to benefit from a wide spectrum of new results ranging from improved diagnostics over vaccine development to better satellite-generated ecological data. 

Why did schistosomiasis become such a scourge in the first place? A strong reason is the high concentration of people near rivers and lakes harbouring the intermediate snail hosts, while curative measures could not be contemplated until the parasite had been discovered [8] and its life-cycle demonstrated [9]. Although these findings clarified the epidemiology, effective disease prevention would not be available until the introduction of praziquantel in the 1970s [10]. Promoting sanitation and telling people to avoid lakes and streams in rural areas without infrastructures were as unrealistic then as it is today, so early activities relied primarily on snail control through various molluscicides released into water bodies used by people in the endemic areas [11] and environmental management (useful only for the type of snail transmitting *S. japonicum*). After the 1980s, however, mass drug administration (MDA) with praziquantel started to make inroads and although reinfection remained a problem, severe morbidity could now be managed through repeated drug treatments. Indeed, chemotherapy proved so efficient that it soon became the only approach contributing to WHO’s 1984 recommendation that morbidity control be the main goal [12], a strategy that is still largely in place. 

Different countries need different approaches since large epidemiological differences between them exist, not the least due to the intermediate sail host, which makes it impossible to apply the same approach everywhere. Although the scope for elimination of schistosomiasis is promising in some countries, the People’s Republic of China (PR China), Brazil, Egypt, Morocco and Oman among them, the situation is altogether different in sub-Saharan Africa, which currently harbours 92% of all schistosomiasis in the world [13]. Thus, research gaps and outstanding issues still remain, one of which is the highly unsatisfactory situation of depending on a single drug for the control of a major disease, even if there is still no evidence of widespread resistance. Equally disconcerting is the realization that the number of people with schistosomiasis will continue to rise as a reflection of the ongoing population growth, while the proportion of people actually receiving drug treatment lingers far behind that of those requiring it [14]. 

## 2. Can Schistosomiasis be Eliminated?

The special issue Prospects for Schistosomiasis Elimination was conceived in response to the need for an all-round view of the present situation with special reference to the tools needed to reach the goal of elimination of one of the worst NTDs that may have caused more human suffering than any other parasitic disease after malaria. Schistosomiasis elimination in PR China has a 60-year history. Repeating this achievement, briefly described in the first paper in this volume [15], in other countries will require long-term governmental support and the incorporation of social and economic approaches boosting local economies and global health development.

Secor and Colley [16] feel that elimination may well be currently feasible in some endemic countries where there have been improvements in sanitation and access to clean water, but point out that sub-Saharan Africa, this strategy switch remains premature. They emphasize the need to develop and evaluate approaches for achieving and validating elimination, as well as defining the level of infection at which a stepped-up approach to interruption of transmission would be feasible. These ideas are echoed by Krauth et al. [17] who discuss possible hidden drivers of transmission that might hamper intervention success and sustainability. They propose a holistic research approach integrating classical epidemiology with modern knowledge of biology, ecology and the social sciences to uncover processes that indirectly influence exposure and transmission. Addressing persisting disease hotspots and neglected population groups could help overcome barriers holding us back from achieving schistosomiasis elimination. An opinion paper by Olveda and Gray [18] emphasizes that the integrated measures pioneered by PR China cannot be duplicated in the Philippines because of the geographical diversity and topography of this country, and the fact that transmission is not seasonal as in PR China. In fact, the situation in the Philippines is more like that of sub-Saharan Africa plus the added problem of dealing with zoonotic transmission. For that reason, they feel an innovative, multi-component approach is critical for long-term sustainable schistosomiasis control and eventual elimination in the Philippines. The key is to ensure high praziquantel coverage of endemic populations and realize the role of bovines in transmission. The bovine population in the endemic areas must either be rigorously treated, vaccinated or replaced with mechanized tractors (as in PR China). Another, more general, opinion paper by Weber et al. [19] highlights the opportunities for public–private partnerships (PPPs) to play a key role in the elimination of schistosomiasis. They point out the critical need for diversified therapeutics and they note gaps in the vaccine and diagnostic product R&D pipelines. 

## 3. Progress Vis-à-Vis the Distribution of Schistosomiasis 

Asian schistosomiasis is zoonotic, which is a cause of concern as successful elimination not only requires management of the human definitive host, but also the animal reservoir hosts. Within Asia, three species affect humans (*S. japonicum*, *S. mekongi* and *S. malayensis*). Most of the published research has focused on *S. japonicum* with comparatively little attention paid to *S. mekongi* and even less to *S. malayensis*. In this special issue, Gordon et al. [20] examined all three species, highlighting the prospects for elimination and current occurrence in their various endemic countries: Cambodia, Lao People’s Democratic Republic (Lao PDR), Malaysia, Myanmar, Thailand, Indonesia, PR China and the Philippines. Apart from the spectacular advancements made in PR China, briefly discussed in the paper by Chen et al. [15], progress in areas and countries, such as the Caribbean, Gabon and Lao PDR, are presented in some detail in this special issue. Excellent progress has been reported previously from Morocco, Africa’s Mediterranean coast and Oman [13], but is not further discussed here. 

Schistosomiasis in Lao PDR was first reported 60 years ago and 10 years later in Cambodia. The infection there is caused by a specific species, *S. mekongi*, which is distributed along a limited part of the Mekong River. Control activities based on mass drug administration resulted in strong advances reducing prevalence to less than 5% according to stool microscopy [21]. Even so, the true number of infected people is probably higher than that reported and further progress will depend on interruption of transmission. Although this type of well-characterized setting offers an exemplary potential for elimination, the local topography, reservoir animals, and a dearth of safe water sources make transmission control a challenge. On the other hand, these limited endemic foci would indeed be excellent testing grounds for what it takes to keep prevalence permanently low.

In the Caribbean, here chronicled by Hewitt and Willingham [22], elimination of schistosomiasis appears achievable in the near term. Transmission has already been eliminated on three islands with six more awaiting official verification. Saint Lucia is now the remaining island with clear evidence of ongoing transmission. Although mass treatment together with snail control using environmental, chemical and biological methods along with public service improvements are important elements in achieving and sustaining elimination, the economic switch from sugarcane and banana production to tourism obviously played a major role.

Mintsa Nguema et al. [23] report the results of mapping the prevalence of schistosomiasis in Gabon (primarily caused by *S. haematobium*), together with the situation with regard to soil transmitted helminthiases (STH) in the northern and eastern regions of the country, which covers 12% of the population. While many STH infections were found to be very common, schistosomiasis prevalence was only 1.7% across the two regions investigated, with no significant difference between them. However, the combined schistosomiasis/STH rate was shown to reach 56.6% and the conclusion was to recommend an integrated approach using praziquantel and mebendazole, supplemented with levamisole due to the high prevalence and intensity of *Trichuris* infections.

## 4. Risk for Fragmented Treatment Coverage

The world burden of schistosomiasis remains considerable, and factors influencing intervention coverage are important. To cover all needs with respect to chemotherapy in a country or region can be difficult, even if the drug and means for its distribution are available. The papers collected here highlight three such situations, though there may well be others, both different and even perhaps more serious. Coverage of praziquantel is an important research area since breaking the transmission of schistosomiasis may remain unattainable without a thorough understanding of this issue.

Adriko et al. [24] carried out a cross-sectional household surveys in an endemic district in Uganda where half the entire population reported never having taken praziquantel. It was found that odds improving use of the drug includes school enrolment and stable settlement in a village. The most frequent reasons that prevented treatment during the latest MDA for a person was either not being offered the drug even if present (49.2%) or being away when it was offered (21.4%). Contrary to expectations, chronically-untreated individuals were rarely systematic non-compliers but rather people who, for various reasons, had repeatedly not been offered treatment.

Coulibaly et al. [25] studied the reasons for low-treatment coverage rates in two endemic villages in Côte d’Ivoire. Based on a questionnaire survey, they found a considerable inter-village heterogeneity (27.7% versus 52.3%) that turned out to be multifactorial. The main reason was work-related (agricultural activities), but the bitter taste of praziquantel and previous experiences with adverse events also played an important role. More than three-quarters of those interviewed who had taken praziquantel declared that they would not participate in future treatments. Therefore, careful consideration should be given to attitudes and practices. 

Students at the college and university level, a group that normally falls outside the common type of surveys, were investigated in Kenya with regard to schistosomiasis and STH infections by Korir et al. [26]. In a sample of close to 300 persons, each tested by three stools/two slides, *S. mansoni* prevalence was found to be 17.8% with 2.5% having heavy *S. mansoni* infections (≥400 eggs/gram faeces). The STH prevalence was much lower. While one wonders why young adults in higher education do not look after themselves better, it is obvious that this group of the population is as approachable for the national schistosomiasis control programmes, as are school children.

## 5. New Approaches

The special issue includes many papers outlining new thoughts, such as geospatial techniques, alternative treatments, high-definition diagnostics and vaccine development. For example, the launch of the Soil Moisture Active Passive (SMAP) satellite in 2015 and the new ECOSTRESS instrument launched in 2018 permit the collection of data that define the ecosystems that govern vector sustainability considerably better than was previously possible, while the high spatial resolution available opened for studies at very specific areas, such as the sub-village, even household, level [27]. This will help delineation of the exact possible geographical distribution of schistosomiasis, including changes related to climate change, through the ecological niche requirements by its intermediate host. 

It is becoming increasingly clear that praziquantel on its own has insufficient activity against transmission since it only affects mature schistosomes Although several doses of praziquantel over a period of 1–2 months would eventually kill all parasites including the initially refractory young ones as they mature during this time. Use of combined artemether/praziquantel, on the other hand, would have an instantaneous effect as artemether would contribute by wiping out the new, still immature generation in the host, as pointed out by Bergquist and Elmorshedy [28]. However, this approach must be limited to areas without malaria co-endemicity to avoid the risk of the development of resistance against artemether, a key drug against malaria. 

## 6. Diagnostics

Low infection intensities are now common thanks to long-term, widespread preventive chemotherapy. Surveillance in this situation requires considerably more sensitive diagnostics than stool microscopy, which is still the standard approach in the endemic areas. Indeed, overlooked low-intensity infections and the fact that praziquantel does not kill all schistosomes in the body, conspire to keep transmission ongoing. 

While diagnosis based on the schistosome circulating cathodic antigen (CCA) [5,29] is clearly more sensitive than stool examination, it does not represent the first high-definition diagnostic technique to be applied for schistosomiasis. Molecular approaches, such as the loop-mediated isothermal amplification (LAMP) [30] and the polymerase chain reaction (PCR) [31], have both been used earlier. The detection of schistosome components based on molecular techniques provide high specificity and high sensitivity, as well as detection of pre-patent infections, and are therefore essential at the elimination stage. Weerakoon et al. present all the various diagnostic techniques in great detail in this special issue [32]. While the assays discussed are similar with respect to sensitivity and can be applied to both blood and faeces, the circulating antigens are also excreted with the urine, which is an advantage that should improve compliancy of continued testing over long periods of time.

## 7. Vaccine Development

Work on schistosomiasis vaccines remains in the doldrums due to the difficulties in convincing donors of the need for a vaccine. The lack of funding for schistosomiasis vaccine development has been debilitating; not that vaccination in itself is a cure-all solution, but even a partially effective vaccine would be a long-term complement to effective chemotherapy. Against all odds, a few candidate molecules for human use have reached the clinical-trials level. One of these is the Sm14/GLA-SE schistosomiasis vaccine, primarily developed against *S. mansoni*, but which cross-reacts with *S. haematobium* and *S. japonicum*, has successfully completed Phase I and Phase IIa clinical trials, with Phase II/III trials underway in Senegal and further trials planned for Brazil. The paper by Tendler et al. [33] in this special issue reviews the development of this vaccine candidate based on the fatty acid-binding protein (FABP) and formulated with the glucopyranosyl lipid A (GLA-SE) adjuvant. The paper follows the development of this product from the initial experiments delivering strongly supportive data to the ongoing Phase II studies. 

Studies undertaken in PR China [34] and the Philippines [35] have identified bovines as major reservoir hosts for *S. japonicum*, and so have provided the rationale for a veterinary-based transmission-blocking vaccine targeting bovines in these settings, benefitting animals directly and humans indirectly. The SjCTPI vaccine [36] has shown promise in experimental trials, and field trials have recently been completed in PR China and the Philippines [37,38]. Interestingly, as shown by You et al. [39] in this special issue, this kind of vaccine would also be useful in African countries although not for humans, only for animals. This work covers the whole field of veterinary schistosomiasis vaccines from irradiated larvae to the recombinant DNA technology revolution. Future challenges to be overcome to design and deliver effective anti-schistosome vaccines are summarized. The fact that schistosomiasis can cause significant animal morbidity and mortality, both in Asia and Africa, is an overlooked cause of economic loss. Although schistosome species, such as *S. bovis, S. curassoni* and *S. mattheei*, do not infect humans, they do infect a wide range of domestic animals, resulting in low economic output, reduced productivity and poor reproduction [40]. Furthermore, the ability of *S. bovis* to form interspecific hybrids naturally with the human parasite *S. haematobium* which has been observed in West Africa [41] should be noted as it presents the potential for zoonotic transmission also in Africa.

A vaccine complemented by praziquantel would not only reduce morbidity for the long term, but also reduce transmission, something that is notoriously difficult to achieve. While vaccination of cattle and water buffaloes can be successful in reducing the risk for human (and bovine) infection in an zoonotic environment, elimination of schistosomiasis in Africa may never be achievable without a human vaccine. However, even if good protection can be shown, full validation, large-scale production and release of a product for human use is still a long time away.

## 8. Research on the Snail 

Efforts to control schistosomiasis started with molluscicides against the snail host and largely ineffectual medicines with serious side effects, vividly described by Jordan [42]. As mentioned above, once introduced on a large scale in the 1980s, the drug praziquantel was so effective that snail control was abandoned for morbidity control. We are now witnessing the return of snail control, here reflected by the submission of as many as four snail-related papers. Two of them deal with the genetic diversity of snails providing findings on the complex biology of this vector, which can be used to define snail phylogeography [43] or to study the molecular basis of snail susceptibility to schistosome infection [44]. The two other papers have completely different foci, one discusses the potential influence of climate change on the distribution of snails capable of transmitting schistosomiasis [45], while the other deals with highly sensitive schistosome diagnosis of infection with the snail based on loop-mediated isothermal amplification (LAMP) [46]. While the very nature of climate change makes the former paper speculative, the latter presents a validated approach to accreditation of schistosomiasis eradication (see below), something of great practical importance. 

## 9. Accreditation

So far, only two infections, both viral, have successfully been eradicated: smallpox in 1979 [47] and cattle rinderpest in 2010 [48]. Rigorous investigations and specifications were required before these diseases were finally eradicated (the viruses still remain in a few high-security laboratories). However, the discussion before the announcements led to an exact definition of the terms “elimination” and “eradication”. The former is defined as the “reduction to zero of the incidence of infection (or to a very low defined target rate) caused by a specified agent in a defined geographical area as a result of deliberate efforts”, while the latter is defined as the “permanent reduction to zero of the worldwide incidence of infection caused by a specific agent as a result of deliberate efforts”. While elimination requires continued measures to prevent re-establishment of disease transmission, intervention measures are no longer needed in the case of eradication. 

The stated aim to eliminate schistosomiasis must rely on considerably more sensitive assays than stool microscopy [4]. It is also important not to forget snail diagnostics. A very important outcome of this special issue is that some of its papers show that we now have the tools needed for the detection of the smallest number of schistosomes in the definitive host (CAA, qPCR or LAMP), as well as in the intermediate snail host, i.e. sporocysts and cercariae (PCR or LAMP). It should, however, be noted that long-term elimination is only possible if neither the intermediate nor the definitive hosts keep the *Schistosoma* life cycle moving as was achieved, and remains the case, in Japan. In addition, regular surveillance would be required, as well as control for potential infections in visiting people.
